# Two-Photon Photodynamic Therapy by Water-Soluble Self-Assembled Conjugated Porphyrins

**DOI:** 10.1155/2013/125658

**Published:** 2012-12-27

**Authors:** Kazuya Ogawa, Yoshiaki Kobuke

**Affiliations:** ^1^Graduate School of Materials Science, Nara Institute of Science and Technology, 8916-5 Takayama, Ikoma, Nara 630-0101, Japan; ^2^Interdisciplinary Graduate School of Medicine and Engineering, University of Yamanashi, 4-3-11 Takeda, Kofu, Yamanashi 400-8511, Japan; ^3^Institute of Advanced Energy, Kyoto University, Gokasho, Uji, Kyoto 611-0011, Japan

## Abstract

Studies on two-photon absorption (2PA) photodynamic therapy (PDT) by using three water-soluble porphyrin self-assemblies consisting of ethynylene-linked conjugated *bis* (imidazolylporphyrin) are reviewed. 2PA cross-section values in water were obtained by an open aperture Z-scan measurement, and values were extremely large compared with those of monomeric porphyrins such as hematoporphyrin. These compounds were found to generate singlet oxygen efficiently upon one- as well as two-photon absorption as demonstrated by the time-resolved luminescence measurement at the characteristic band of singlet oxygen at 1270 nm and by using its scavenger. Photocytotoxicities for HeLa cancer cells were examined and found to be as high as those of hematoporphyrin, demonstrating that these compounds are potential candidates for 2PA-photodynamic therapy agents.

## 1. Introduction

Photodynamic therapy (PDT) is a gentle treatment modality for cancers based on the localization of a photosensitizer such as Photofrin (a mixture of hematoporphyrin oligomers) in the cancer cell followed by photoactivation [1]. In the photoreaction, the photosensitizer is promoted by photoirradiation to the excited triplet state from the excited singlet state through intersystem crossing (ISC) and transfers the excited energy to ground state oxygen (^3^O_2_) generating the singlet oxygen (^1^O_2_) which destroys the cancer. One of the problems in current PDT is the limitation of the penetration depth of light at 630 nm to restrict the treatment of deep cancer. However, the absorption by tissues is much lower in the near-infrared (NIR) region between 700–1300 nm, which is called as an optical window of biological tissue [2]. Thus, the use of light in the NIR enables the deep part cancer treatment.

Two photon absorption (2PA) is a nonlinear optical process, in which two-photons are absorbed simultaneously at wavelength practically in the NIR region even where no one-photon absorption exists to promote a molecule to the excited state corresponding to the combined energy of the two-photons. Moreover, the quadratic dependence of 2PA on the laser intensity allows a high spatial selectivity by using a focused laser beam. Therefore, PDT using 2PA is better for treating the deeper cancer with a three-dimensional selectivity. In 1990s, the two photon absorption photodynamic therapy (2PA-PDT) has been proposed and studied by some research groups [3–7]. However, these studies could not attract a lot of attention because 2PA efficiencies of photosensitizers used in those studies were low with 2PA cross-section values below 50 GM (1 GM equals to 10^−50^ cm^4^ s molecule^−1^ photon^−1^). For example, the 2PA cross-section value of protoporphyrin IX is known to be only ~2 GM [8] and other organic molecules also exhibited small values less than 1000 GM measured by femtosecond pulses. Photofrin was also investigated as a candidate for 2PA-PDT [9]. The *σ* ^(2)^ value of Photofrin was determined as 7.4 GM at 850 nm, and they conducted cell experiments. The total energy required for the 50% cell death was 6,300 J cm^−2^, which required 4 hours irradiation, demonstrating that Photofrin was unsuitable for 2PA-PDT, and new sensitizers having much higher *σ* ^(2)^ values would be requested. After that, we [10–12] and some research groups [13–16] have reported 2PA-PDT studies using photosensitizers with much higher *σ* ^(2)^ values. 2PA-PDT employing energy transfer from a two photon absorbing dye having the *σ* ^(2)^ value of 217 GM to pheophorbide as a PDT photosensitizer was reported [13]. In this case, the 2PA-PDT effect was observed when two photon irradiation of Hela cells was treated overnight. Anderson reported* in vitro* 2PA-PDT as well as closure of blood-vessel by two photon excitation of butadiynylene-connected zinc-porphyrin dimer [14].

**Scheme 1:**
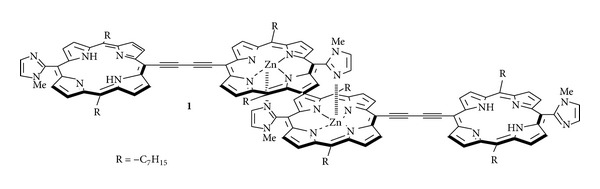
Supramolecular porphyrin array **1**.

In 2003, we reported that the self-assembled conjugated porphyrin **1** (Scheme 1) through zinc-imidazolyl coordinations exhibiting a large two photon absorption cross-section value (*σ* ^(2)^) of 7,600 GM, which was the largest among the reported values measured using femtosecond pulses [16, 17]. This value is three or four orders of magnitude larger than that of protoporphyrin IX or Photofrin. Further, **1** was found to generate singlet oxygen with high efficiency in toluene, indicating an appropriate candidate for 2PA-PDT. Thus, we started the 2PA-PDT study with the water-soluble conjugated porphyrins. In this paper, we will report on our recent studies on the 2PA-PDT, including the syntheses of water-soluble porphyrin self-assemblies, their two photon absorption properties, singlet-oxygen generation, and photocytotoxicity.

## 2. The First 2PA-PDT System Based on Self-Assembled Porphyrin Array 1 [10]

**Scheme 2:**
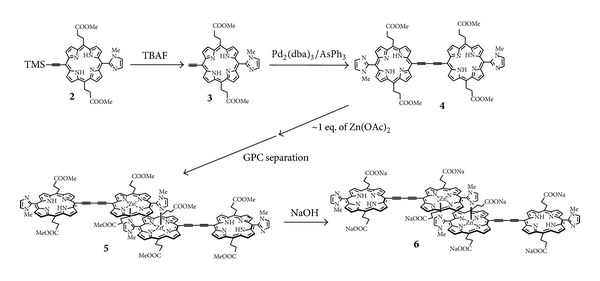
Synthetic routes of water-soluble butadiyne-linked self-assembly **6**.

In order to solubilize porphyrin **1** in water, methoxycarbonylethyl groups, which would be hydrolyzed to give carboxyl groups, were introduced instead of heptyls at two *meso*-positions in each porphyrin. As shown in Scheme 2, *bis*(imidazolylporphyrin) **4** bridged by a butadiyne linkage was synthesized from TMS-deprotected compound **3** by a Pd(0)-mediated coupling reaction with 47% yield. The reaction of **4** with one equivalent of zinc acetate gave a complementary dimer of monozinc-*bis*(imidazolylporphyrin) **5**, which was isolated using gel permeation chromatography (GPC). Finally, the methyl ester groups were hydrolyzed by sodium hydroxide to obtain water-soluble self-assembly **6**.

**Scheme 3:**
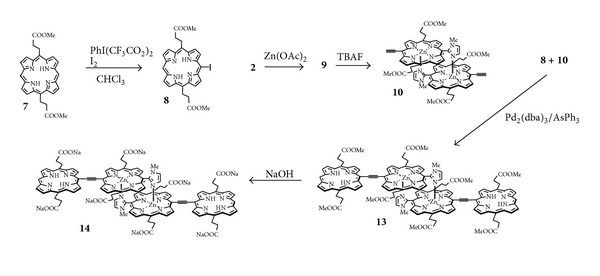
Synthetic routes of water-soluble monoacetylene-linked self-assembly **14**.

The low yield of **6** (~12%) was attributed to the monometalation process, giving a mixture of starting *bis*(free base) porphyrin **4**, the desired monozinc complex **5**, and dizinc complex. The yield was further considerably decreased during GPC separation. The low yield is obviously disadvantageous for the practical use. In order to improve this problem, we designed a monoacetylene-linked self-assembly. In this case, the one-step heterocoupling reaction of monomeric zincporphyrin with free base porphyrin can be employed to produce directly the desired monoacetylene-linked, monozinc-freebase bis-porphyrin. As shown in Scheme 3, for the heterocoupling reaction, acetylenic porphyrin **10** was prepared from porphyrin **2** by zinc insertion, followed by deprotection of the TMS group. The starting porphyrin **7** was iodinated with PhI(CF_3_CO_2_)_2_ and iodine to give the coupling counterpart **8**. The heterocoupling reaction of **8** and **10** was conducted using Pd_2_(dba)_3_/AsPh_3_ as a catalyst system similar to the synthesis of **4**. The ester hydrolysis of **13** was performed to allow **14** in a manner similar to the case of **6**. The total yield of **14** was ~40% based on monomer **7**, significantly improved compared to that of **6**.

**Figure 1:**
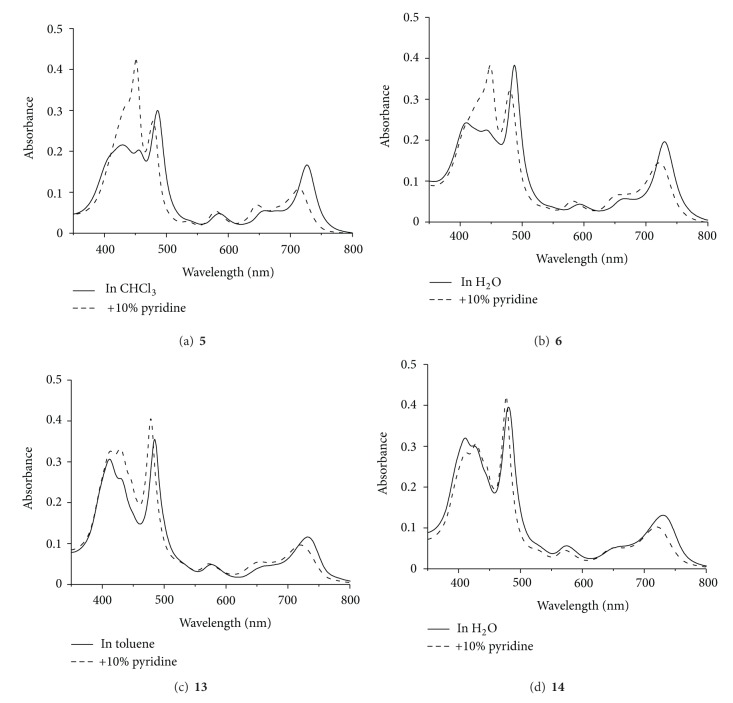
UV/Visible absorption spectra; (a) **5** in CHCl_3_ (solid line) and in CHCl_3_/pyridine (dashed line), (b) **6** in H_2_O (solid line) and in H_2_O/pyridine (dashed line), (c) **13** in toluene (solid line) and in toluene/pyridine (dashed line), and (d) **14** in H_2_O (solid line) and in H_2_O/pyridine (dashed line). All concentrations were adjusted to ca. 0.5 *μ*M.

The absorption spectrum of ester form **5** in chloroform is shown in Figure 1(a) (solid line). The Soret band and the Q-band were observed at 486.5 and 726.5 nm, respectively. After adding 10% pyridine that can cleave the complementary coordination of imidazolyl to zinc, these two peaks were blue-shifted to 478.5 and 714.5 nm, respectively (bold line), suggesting dissociation to monomeric bis-porphyrin by the disappearance of the head-to-tail type exciton interaction between two bis-porphyrins. Similar spectral changes were observed for **6** in water (Figure 1(b)), demonstrating that **6** existed as the self-assembled dimer in water by the complementary coordination in contrast to the monomeric form in the presence of 10% pyridine. The similar behavior was observed for ester form **13** and its water-soluble **14** (Figures 1(c) and 1(d)), also indicating that the dimer structure is maintained for **14** in water.

**Figure 2:**
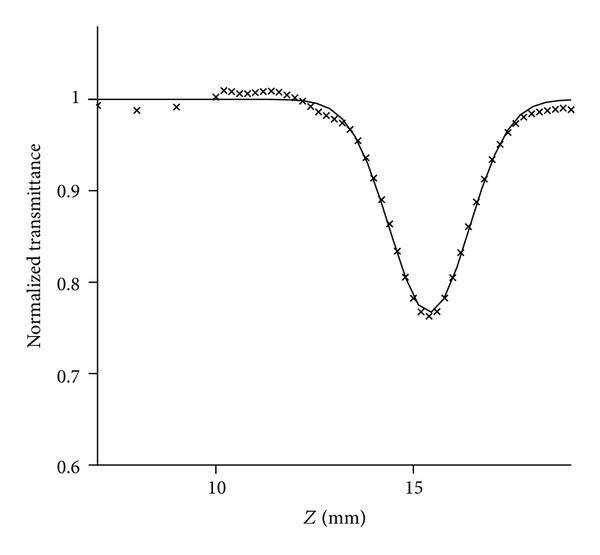
Typical open-aperture Z-scan trace (×) of **6** in water.

The *σ* ^(2)^ values of **6** and **14** in water were measured by an open aperture Z-scan method at 850 nm with 150 fs pulses.  Figure 2 shows a typical open-aperture Z-scan trace (×) of **6**. The detailed experimental conditions were described in [10]. The *σ* ^(2)^ values were determined as 7,500 for **6** and 7,900 GM for **14**, respectively. The value of 7,500 GM obtained for **6** in water is equivalent to that of **1** in chloroform, indicating no solvent effect on *σ* ^(2)^ values. These two values are almost identical, suggesting that the 2PA efficiency of monoacetylene bridge is equivalent to that of bisacetylene. The values are significantly large compared to those of protoporphyrin [8] and hematoporphyrin [9], indicating that **6** and **14** are possible candidates for the 2PA-PDT agent.

**Figure 3:**
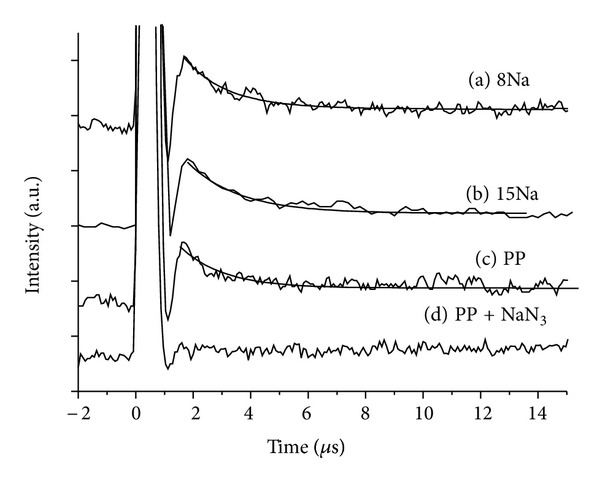
Time-resolved emission profiles at 1270 nm in H_2_O; (a) **6**, (b) **14**, (c) protoporphyrin (**PP**), and (d) **PP** with NaN_3_.

**Figure 4:**
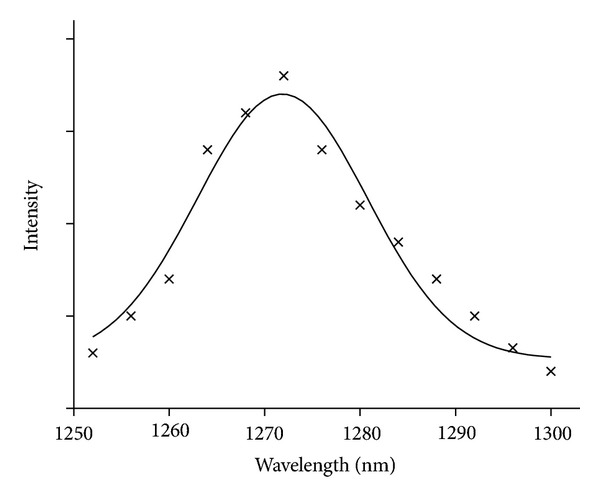
Typical emission spectrum of singlet oxygen in H_2_O solution of **PP**.

The major pathway in PDT is generally accepted as the Type-II reaction associated with singlet oxygen generation which involves energy transfer from the triplet state photosensitizer to ground state oxygen to give toxic singlet oxygen (^1^O_2_*) that attacks tumor cells. Thus, the efficient generation of singlet oxygen in water is required for PDT agents. The direct evidence for singlet oxygen generation can be monitored by phosphorescence from ^1^∆_*g*_ to ^3^Σ_g_ at 1270 nm. The emission from the singlet oxygen at 1270 nm under one-photon irradiation condition was measured by time-resolved experiment. The agents in water were irradiated by non-focused 5 ns Nd:YAG-OPO pulses (10 Hz and 128 shots) with a pulse energy of around 3 mJ, and the phosphorescence was detected through an interference filter with an InP/InGaAsP detector operated at −80°C. The sample concentration was 5 × 10^−5^ M, and the excitation wavelengths were selected to be the same absorbance (0.8) at 556 nm for **6**, and protoporphyrin (PP), and at 550 nm for **14**. The detailed experimental conditions were also described in [10].  Figure 3 shows time-resolved emission profiles at 1270 nm for (a) **6**, (b) **14**, (c) **PP**, and (d) **PP** with NaN_3_ as the quencher. The fast rise components were observed for (a), (b), and (c) after the excitation, suggesting the formation of singlet oxygen by energy transfer from the photosensitizer. The lifetime was determined as ~2 *μ*s, which was similar to the reported value of singlet oxygen in water (1.5 ~ 4 *μ*s) [19–22]. NaN_3_, being a quencher for singlet oxygen, was added into all the solutions of **6**, **14**, and **PP** to quench the emission, demonstrating that the emission originated from singlet oxygen (typical data were shown in Figure 3(d)). Moreover, as shown in the emission spectrum (typical spectrum of **PP** was presented in Figure 4) recorded in the range from 1250 to 1300 nm with the same equipment using a monochromator (the intensity was obtained by integrating decay profile from around 2 to 8 *μ*s), the spectral shape with a peak maximum at around 1271 nm is similar to that for singlet oxygen as reported in the literature [20]. Samples of **6** and **14** showed almost the same emission intensity, time profile, and spectrum to **PP**.

**Figure 5:**
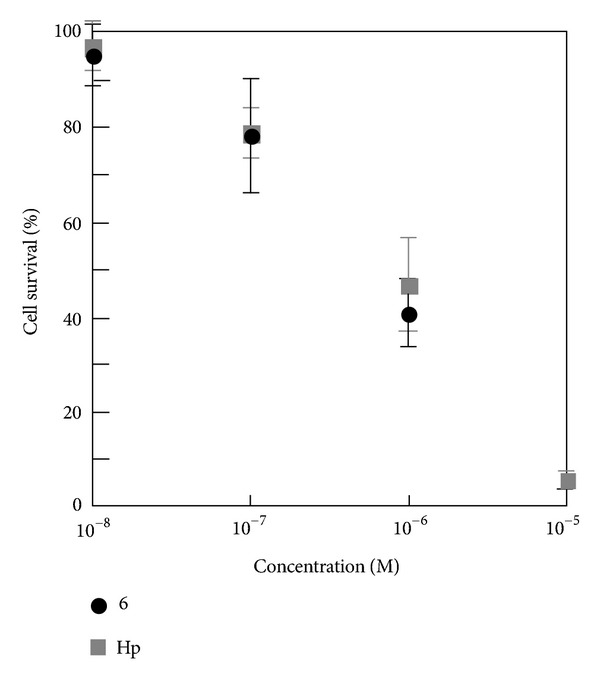
Photocytotoxicity of **6** and hematoporphyrin (Hp) for HeLa cell.

The photocytotoxicity of the agents was examined using HeLa cells under one-photon irradiation conditions.  Figure 5 shows the photocytotoxicity of **6**. The cell survival percentages after the photoirradiation was plotted against the concentration of agents. The cell was almost unchanged for concentrations lower than 10^−8^ M and cell survival decreased with increasing agent concentration. No significant difference in the photocytotoxicity was observed between **6** and hematoporphyrin (Hp), demonstrating that **6** exhibits high PDT efficiency equivalent to Hp.

**Figure 6:**
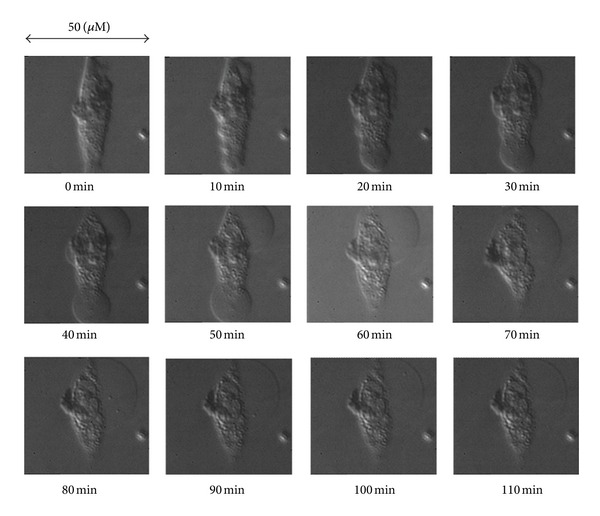
Time course of cell dying for HeLa cell upon photoirradiation with **14** (5 × 10^−6^ M).

The photocytotoxicity of **14** for HeLa cell was also examined by observing cell death upon photoirradiation using a microscope. A CW diode laser (671 nm) was used for excitation. The spot diameter was 30 *μ*m with a power density of 1.8 W/cm^2^, and the beam center was adjusted at the center of cell. The details were described in [10]. No cell death was observed without the agent, even after 2 hours of irradiation (total irradiation energy >12,960 J/cm^2^ with a power density of 1.8 W/cm^2^). On the other hand, cell death was observed by administrating **14** (5 × 10^−6^ M). As shown in Figure 6, the leakage of the cytoplasm worsened with time course, and blebs were formed on the cell surface.  Table 1 summarizes the irradiation time required for cell death at various concentrations of **14**. The exposure time until cell death was shortened with increasing concentrations of **14**. These results demonstrate that water-soluble porphyrin self-assemblies **6** and **14** are potential candidates for 2PA-PDT.

## 3. The Second 2PA-PDT System Using Dendritic-Type Substituent [11, 12]

**Scheme 4:**
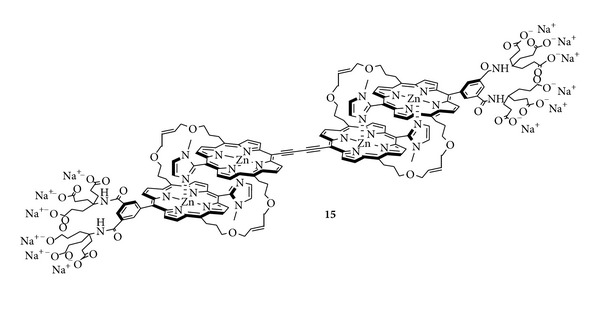
Water-soluble supramolecular porphyrin **15**.

Next, we reported a different approach to construct a water-soluble two photon absorbing porphyrin-based photosensitizer **15** (Scheme 4) as another potential candidate for 2PA-PDT. A butadiyne-bridged bis-porphyrin was chosen as the two photon absorbing part of this 2PA-PDT system. In contrast to the previous compounds, a dendritic-type substituent was used as hydrophilic groups. A monomeric porphyrin having six carboxylates was attached at both ends of the butadiyne-bridged bis-porphyrin through zinc-imidazolyl coordination to allow a tetramer. The self-assembled structure was covalently fixed by olefin metathesis. [22]. In contrast to the previous compounds, the hydrophilic groups in compound **15** were larger in number and were located only at both ends of the tetramer. These factors may affect drug-delivery property into the cell.

**Scheme 5:**
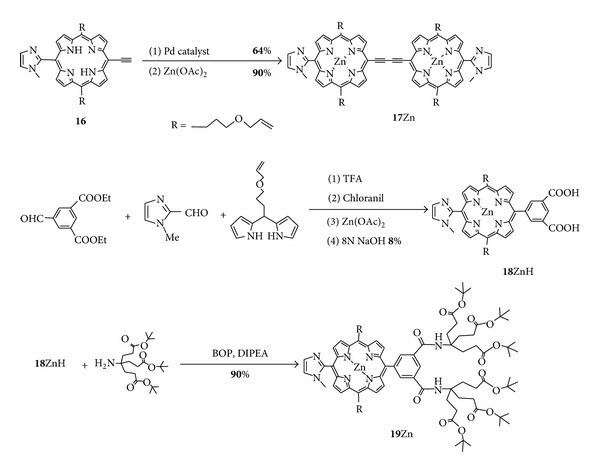
Synthetic routes of **17**Zn and **19**Zn.

Scheme 5 shows synthetic routes of a zinc-inserted butadiyne-bridged imidazolylporphyrin dimer **17**Zn as the 2PA component and a zinc-inserted isophthalamidoimidazolylporphyrin having 12 carboxylic acid groups **19**Zn as the water-soluble component. 

The butadiyne-bridged bis-porphyrin was synthesized by Pd-catalyzed coupling of **16** using Pd_2_(dba)_3_ (dba = dibenzylideneacetone) and triphenylarsine to afford freebase **17** in 64% yield. Freebase **17** was treated with zinc acetate to give the 2PA component **17**Zn. In order to increase hydrophilicity, the water-soluble component **19**Zn was synthesized from **18**ZnH with a precursor of dendrimer via BOP (benzotriazol-1-yloxytris(dimethylamino)phosphoniumhexafluorophosphate) condensation in a 90% yield.

**Scheme 6:**
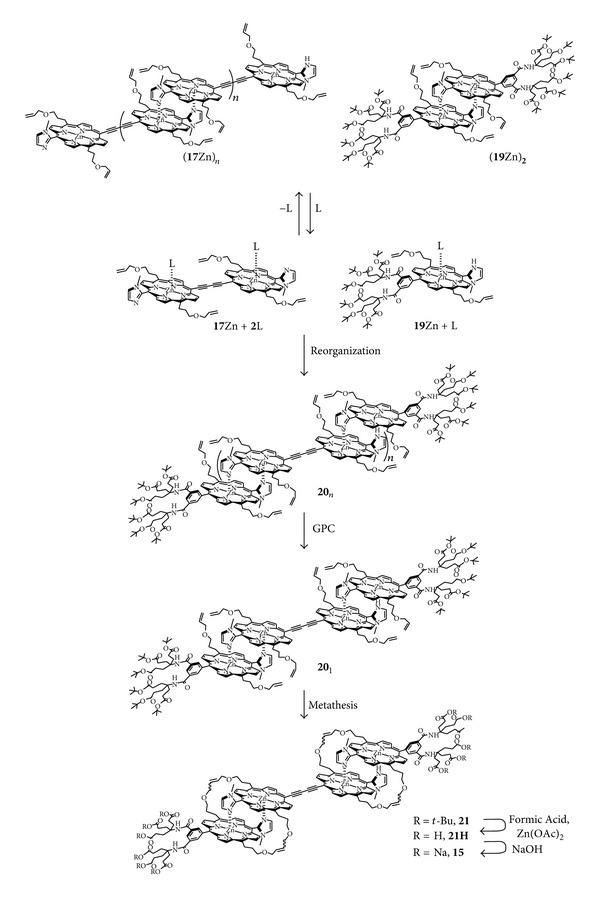
Synthetic routes of **15**.

In noncoordinating solvents such as CHCl_3_, imidazolylporphyrins **17**Zn, and **19**Zn exist as polymer (**17**Zn)_n_ and as dimer (**19**Zn)_2_, respectively, through the complementary coordination of imidazolyl to zinc as shown in Scheme 6. However, in coordinating solvents such as pyridine (denoted as L in Scheme 6), porphyrins **17**Zn and **19**Zn exist as their monomeric form. In order to lead to the desired tetramer **20**_1_, the initial coordination dimers of **19**Zn and **17**Zn in a 2 : 1 molar ratio were dissociated by dissolving in pyridine. Reorganization was conducted by removing pyridine to form different length arrays of **20**_*n*_. The tetramer **20**_1_ can be isolated using preparative GPC (8.7%). In order to prevent reorganization in other solution conditions, the coordination structure was fixed via metathesis of the allyl ether side chains using Grubbs catalyst to get compound **21** (80%). Compound **21** was treated with formic acid to cleave *t*-Bu groups giving the carboxylic acid form **21**H, and subsequent treatment with an equimolar amount of NaOH yielded the water-soluble tetramer **15** (85%). The characterizations of the compound **15** including GPC, mass, UV/vis absorption and emission spectral measurements were described in detail in [11].

**Figure 7:**
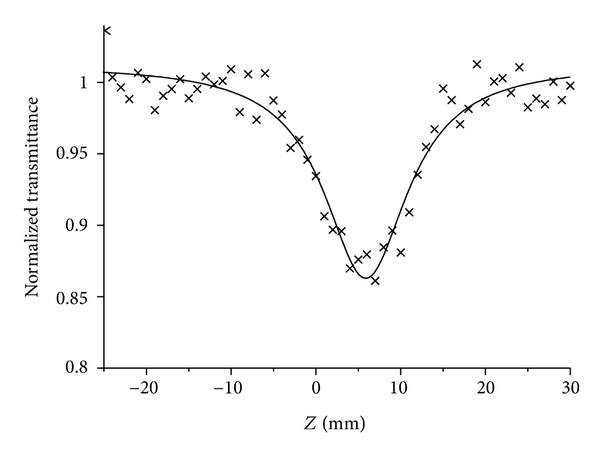
Typical open-aperture *Z*-scan trace (×) of 0.4 mM of **15** in water.

**Figure 8:**
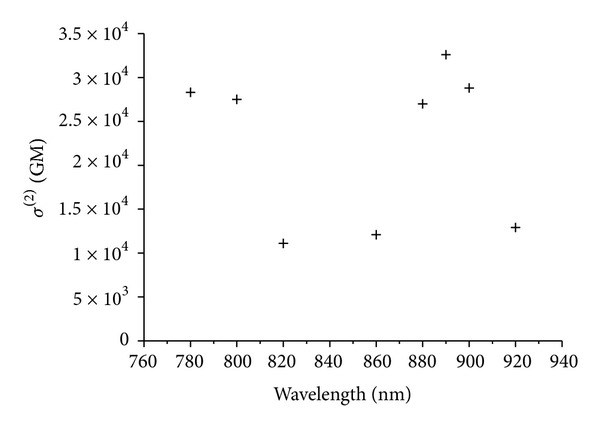
Effective 2PA spectrum of 0.4 mM of **15** in water [11, 18].

The effective 2PA cross-section was measured using an open-aperture Z-scan method with nanosecond pulses. A typical Z-scan trace of compound **15** in water at 890 nm with theoretically fitted curve is shown in Figure 7. The effective 2PA spectrum of compound **15** in water is shown in Figure 8 [11, 18].

The 2PA maximum peak for compound **15** appeared at 890 nm with a value of 33,000 GM. It should be noted that it is difficult to compare this 2PA cross-section value with those of **6** and **14** obtained by femtosecond pulses. The nanosecond values are ca. 30 times larger compared to the femtosecond values for our previously reported compounds [17]. The large discrepancy between nanosecond and femtosecond values is attributed to excited state absorption (ESA) due to the longer pulse width in nanosecond lasers as compared to those in femtosecond pulses. The effective *σ* ^(2)^ value of compound **15** was three orders of magnitude larger than that of H2TPP (29 GM at 780 nm) measured by employing the same nanosecond pulses [20].

**Figure 9:**
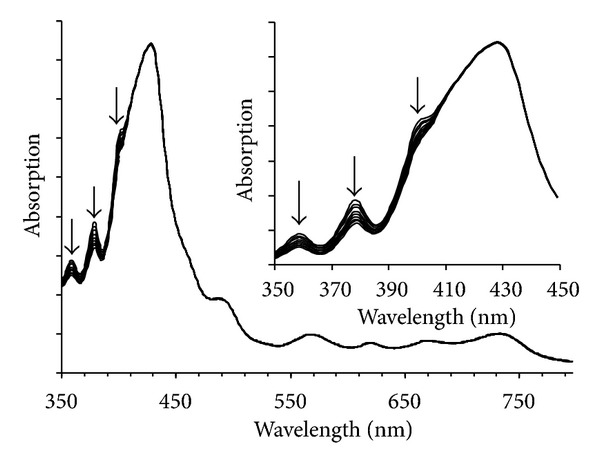
Change in the absorption spectra of ADPA with **15** upon two photon irradiation.

**Figure 10:**
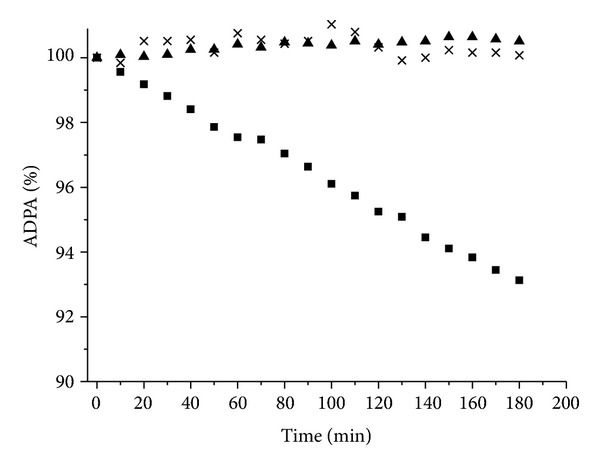
Photobleaching of the ADPA absorption peak at 379 nm (5.0 × 10^−5^ M) after two photon irradiation at 890 nm (no photosensitizer (triangle), TPPS (×) (1.0 × 10^−4^ M), and **15** (square) (5.0 × 10^−5^ M) in D_2_O.

Compound **15** generated singlet oxygen by one-photon irradiation as seen in Figures 3 and 4 [12]. However, this direct measurement could not be applied to the two photon conditions since the emission signal was too weak to detect. Singlet oxygen can not only be measured by the direct observation but also be determined quantitatively by using scavengers such as anthracene-9,10-dipropionic acid sodium salt (ADPA) [11, 23, 24] which reacts with oxygen to form an endoperoxide. Therefore, singlet oxygen generation by two photon irradiation was monitored as decrease in ADPA absorption. ADPA exhibits characteristic absorption peaks at 399, 378, 359, and 342 nm. A D_2_O solution of ADPA and compound **15** was irradiated with focused 100 fs pulses at 890 nm with a pulse energy of 4 nJ corresponding to the peak power of 6.1 GW/cm^2^ [11]. Since the emission from singlet oxygen is very week under the two photon conditions and the lifetime is short in H_2_O, D_2_O was used as solvent [21]. Continuous photobleaching of anthracene absorption was observed for 3 h using 890 nm excitation. On the other hand, no change was observed in the Q-bands of compound **15** indicating that the sensitizer itself was not affected either during two photon irradiation or by singlet oxygen generation (Figure 9). Almost no decrease in the anthracene absorption was observed in the solution without **15**. The same experiment was conducted by using tetraphenylporphyrin tetrasulfonic acid (TPPS) which has very low 2PA efficiency at this wavelength region. The results were summarized in Figure 10 (no photosensitizer (triangle), TPPS (×) and **15** (square)). This indicates that compound **15** is a potential agent for 2PA-PDT. The detailed experimental conditions and data were described in [11, 12].

**Figure 11:**
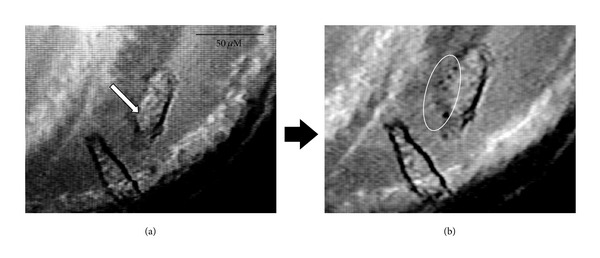
Pictures of HeLa cells incubated with **15** before (a) and after (b) two photon excitation with 100 fs pulses at 780 nm. The irradiated position is marked by a white arrow. The degradation of the cell membrane was observed as indicated by an oval.

Finally, the PDT experiment with two photon irradiation was conducted using HeLa cells. A HeLa cell incubated with **15** on a glass slide was irradiated for 5 min with 100 fs pulses at 780 nm with an average power of 2 mW which provides an average of 600 mJ/cell. Detailed experimental conditions were described in [12]. As shown in Figure 11(a), a HeLa cell at the upper site was selectively excited on the position marked by an arrow. After the irradiation, the degradation of the cell membrane was observed in the upper cell (Figure 11(b)). The lower cell which was nonirradiated was undamaged. Control experiments with Hp and without photosensitizer also resulted in no cell damage. These results suggest that compound **15** is a potential agent not only for photodynamic activity on HeLa cells but also for selective targeting of tumor cells via two photon excitation. Although femtosecond laser sources were not available in the cell experiments of compounds **6** and **14**, it would be interesting to conduct a comparative study between **15** and the previous compounds in order to determine which type of structure and hydrophilicity will give better drug delivery property.

## Figures and Tables

**Table 1:** Photocytotoxicity of **14** for a single HeLa cell (671 nm).

Concentration	Time of cell death*	Irradiation energy
*μ*M	min	J/cm^2^
0	>120	>12960
1	113 ± 12	12204
5	74 ± 9	7992
10	48 ± 3	5184

*Time of cell death was determined by the trypan blue staining method.

## References

[1] Moan J, Peng Q, Patrice T (2004). An outline of the history of PDT. *Photodynamic Therapy*.

[2] Waynant RW, Ediger MN (1993). Lasers in medicine. *Electrooptics Handbook*.

[3] Bhawalkar JD, He GS, Prasad PN (1996). Nonlinear multiphoton processes in organic and polymeric materials. *Reports on Progress in Physics*.

[4] Shea CR, Hefetz Y, Gillies R, Wimberly J, Dalickas G, Hasan T (1990). Mechanistic investigation of doxycycline photosensitization by picosecond-pulsed and continuous wave laser irradiation of cells in culture. *Journal of Biological Chemistry*.

[5] Lenz P (1995). *In vivo* excitation of photosensitizers by infrared light. *Photochemistry and Photobiology*.

[6] Bhawalkar JD, Kumar ND, Zhao CF, Prasad PN (1997). Two-photon photodynamic therapy. *Journal of Clinical Laser Medicine and Surgery*.

[7] Fisher WG, Partridge WP, Dees C, Wachter EA (1997). Simultaneous two-photon activation of type-I photodynamic therapy agents. *Photochemistry and Photobiology*.

[8] Goyan RL, Cramb DT (2000). Near-infrared two-photon excitation of protoporphyrin IX: photodynamics and photoproduct generation. *Photochemistry and Photobiology*.

[9] Karotki A, Khurana M, Lepock JR, Wilson BC (2006). Simultaneous two-photon excitation of photofrin in relation to photodynamic therapy. *Photochemistry and Photobiology*.

[10] Ogawa K, Hasegawa H, Inaba Y (2006). Water-soluble bis(imidazolylporphyrin) self-assemblies with large two-photon absorption cross sections as potential agents for photodynamic therapy. *Journal of Medicinal Chemistry*.

[11] Dy JT, Ogawa K, Satake A, Ishizumi A, Kobuke Y (2007). Water-soluble self-assembled butadiyne-bridged bisporphyrin: a potential two-photon-absorbing photosensitizer for photodynamic therapy. *Chemistry*.

[12] Ogawa K, Dy J, Kobuke Y, Ogura S, Okura I (2007). Singlet oxygen generation and photocytotoxicity against tumor cell by two-photon absorption. *Molecular Crystals and Liquid Crystals*.

[13] Kim S, Ohulchanskyy TY, Pudavar HE, Pandey RK, Prasad PN (2007). Organically modified silica nanoparticles co-encapsulating photosensitizing drug and aggregation-enhanced two-photon absorbing fluorescent dye aggregates for two-photon photodynamic therapy. *Journal of the American Chemical Society*.

[14] Collins HA, Khurana M, Moriyama EH (2008). Blood-vessel closure using photosensitizers engineered for two-photon excitation. *Nature Photonics*.

[15] Dahlstedt E, Collins HA, Balaz M (2009). One- and two-photon activated phototoxicity of conjugated porphyrin dimers with high two-photon absorption cross sections. *Organic and Biomolecular Chemistry*.

[16] Gallavardin T, Armagnat C, Maury O (2012). An improved singlet oxygen sensitizer with two-photon absorption and emission in the biological transparency window as a result of ground state symmetry-breaking. *Chemical Communications*.

[17] Ogawa K, Ohashi A, Kobuke Y, Kamada K, Ohta K (2003). Strong Two-Photon Absorption of Self-Assembled Butadiyne-Linked Bisporphyrin. *Journal of the American Chemical Society*.

[18] Ogawa K, Ohashi A, Kobuke Y, Kamada K, Ohta K (2005). Two-photon absorption properties of self-assemblies of butadiyne-linked bis(imidazolylporphyrin). *Journal of Physical Chemistry B*.

[19] Rodgers MA, Snowden PT (1982). Lifetime of oxygen (O2(1.DELTA.g)) in liquid water as determined by time-resolved infrared luminescence measurements. *Journal of the American Chemical Society*.

[20] Ogilby PR, Foote CS (1983). Chemistry of singlet oxygen. 42. Effect of solvent, solvent isotopic substitution, and temperature on the lifetime of singlet molecular oxygen (1Δg). *Journal of the American Chemical Society*.

[21] Schweitzer C, Schmidt R (2003). Physical mechanisms of generation and deactivation of singlet oxygen. *Chemical Reviews*.

[22] Frederiksen PK, McIlroy SP, Nielsen CB (2005). Two-photon photosensitized production of singlet oxygen in water. *Journal of the American Chemical Society*.

[23] Lindig BA, Rodgers MAJ, Schaap AP (1980). Determination of the lifetime of singlet oxygen in D2O using 9,10-anthracenedipropionic acid, a water-soluble probe. *Journal of the American Chemical Society*.

[24] Oar MA, Serin JM, Dichtel WR, Fréchet JMJ, Ohulchanskyy TY, Prasad PN (2005). Photosensitization of singlet oxygen via two-photon-excited fluorescence resonance energy transfer in a water-soluble dendrimer. *Chemistry of Materials*.

